# Pechiche (*Vitex cymosa* Berteo ex Speng), a Nontraditional Fruit from Ecuador, is a Dietary Source of Phenolic Acids and Nutrient Minerals, in Addition to Efficiently Counteracting the Oxidative-Induced Damage in Human Dermal Fibroblasts

**DOI:** 10.3390/antiox9020109

**Published:** 2020-01-27

**Authors:** Mabel Guevara, Luis A. Valdés-Silverio, María G. Granda-Albuja, Gabriel Iturralde, Tatiana Jaramillo-Vivanco, Francesca Giampieri, Celestino Santos-Buelga, Ana M. González-Paramás, Maurizio Battino, José M. Álvarez-Suarez

**Affiliations:** 1 Grupo de Investigación en Biotecnología Aplicada a Biomedicina (BIOMED). Universidad de Las Américas, Quito 170125, Ecuador; guevarateran@usal.es (M.G.); tatiana.jaramillo@udla.edu.ec (T.J.-V.); 2 Grupo de Investigación en Polifenoles, Universidad de Salamanca, Campus Miguel de Unamuno, 37007 Salamanca, Spain; csb@usal.es (C.S.-B.);; 3 Glendale Community College, Glendale, AZ 85302, USA; lui2152145@maricopa.edu; 4 Laboratorios de Investigación. Universidad de Las Américas, Quito 170125, Ecuador; maria.granda.albuja@udla.edu.ec (M.G.G.-A.); gabriel.iturralde@udla.edu.ec (G.I.); 5 Jardín Botánico de Quito, Quito 170125, Ecuador; 6 Nutrition and Food Science Group, Department of Analytical and Food Chemistry, CITACA, CACTI, University of Vigo, Vigo Campus, 36310 Vigo, Spain; f.giampieri@univpm.it (F.G.);; 7 Department of Clinical Sciences, Universitá Politecnica delle Marche, 60131 Ancona, Italy; 8 College of Food Science and Technology, Northwest University, Xi’an 710069, China; 9 International Research Center for Food Nutrition and Safety, Jiangsu University, Zhenjiang 212013, China; 10 King Fahd Medical Research Center, King Abdulaziz University, Jeddah 21589, Saudi Arabia

**Keywords:** *Vitex cymosa* Berteo ex Speng, pechiche, nontraditional fruits, Ecuador, antioxidant capacity, oxidative stress

## Abstract

Pechiche fruits (*Vitex cymosa* Berteo ex Speng) from Ecuador were studied to determine their phenolic acid profile, nutrient minerals and capacity to protect primary human dermal fibroblasts (HDFa) against oxidative-induced damage. Up to five phenolic acids were identified, with homovanillic acid as the main one. Vitamin C, β-carotene and lutein were also determined. Phosphorus and potassium were the main macrominerals, while iron was the principal micromineral. HDFa were preincubated with a crude pechiche extract (PCext) and then subjected to oxidative stress. The activity of five antioxidant enzymes, intracellular reactive oxygen species (ROS) and ATP levels and lipid peroxidation and protein oxidation were used as markers of oxidative damage. Preincubation with PCext for 24 h allowed for the significant reduction of intracellular ROS levels, improved the intracellular ATP levels and protected lipids and proteins against oxidative damage (*p* < 0.05). Additionally, preincubation with PCext was also able to significantly (*p* < 0.05) improve the activity of the antioxidant enzymes catalase, superoxide dismutase, glutathione peroxidase, glutathione reductase and glutathione transferase, compared to the stressed group without pretreatment. The results obtained in this study suggest the potential of pechiche as a source of bioactive compounds, as well as its beneficial effect against oxidative stress.

## 1. Introduction

Several studies provide scientific evidence that points to the relationship between the occurrence of chronic noncommunicable diseases and the imbalance between levels of free radicals species (mainly reactive oxygen species or ROS) and endogenous antioxidant defenses [1]. In fact, several studies have shown that populations with food patterns rich in fruit and vegetables suffer fewer incidences of chronic degenerative diseases related to aging and oxidative stress [2,3]. In this sense, knowledge of bioactive compounds’ contribution to local diets could allow for a better understanding of these diets’ potential in the prevention of these diseases. Currently, the search for new sources of bioactive compounds has led to researchers becoming interested in exotic and tropical fruits due to their potential as a source of bioactive compounds and their therapeutic value. In this context, traditional and nontraditional fruits consumed by the populations of South America, such as Ecuador, have been of great interest and have been studied in recent times [4,5,6,7,8,9,10,11].

**Figure 1:**
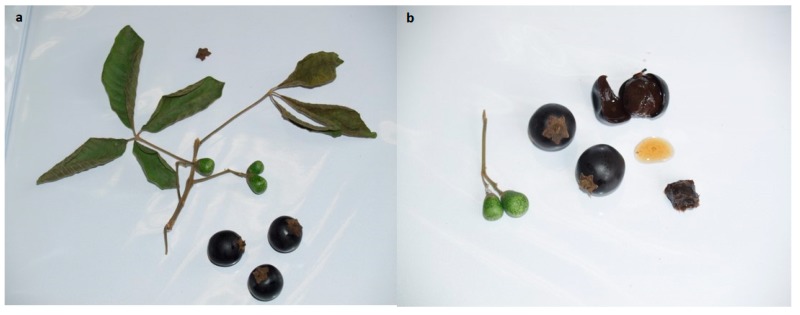
Pechiche fruit. (**a**) Pechiche plant leaves with ripe and unripe fruits. (**b**) Ripe and unripe fruits, epicarp and endocarp, seed and fruit juice.

Ecuador has a variety of climatic conditions with an extensive variety of native and exotic fruit species of potential interest as sources of several nutrients and bioactive compounds. Mainland Ecuador has three well-defined geographical regions characterized by their climatic conditions and typical vegetation. The coast, a lowlands region, is located between the Andes mountain range and the Pacific Ocean and is home to one of the largest production areas of tropical fruits, be they native, exotic or introduced, and these are distributed and consumed nationwide and, in some cases, exported abroad. As part of the typical vegetation of this region, one finds the nontraditional fruit known as pechiche (*Vitex cymosa* Berteo ex Speng). Pechiche belongs to the *Vitex* genus, Lamiaceae family and is a small tree or shrub occurring in tropical and subtropical regions. This plant is endemic to Panama, the Netherlands Antilles, Venezuela, Brazil, Bolivia, Colombia, Ecuador, Peru, Argentina and Paraguay and is threatened due to deforestation [12]. The plant is commonly known as taruma, as aceituno in Colombia or as pechiche in Ecuador. In Ecuador, it is found growing wild in the provinces of Guayas, El Oro, Manabí and Los Ríos, all located in the western coastal region [12]. Pechiche fruits are harvested between December and February and consist of a fleshy drupe of black or purple; they are ovoid between 1.5 and 2 cm in length with a bitter taste (Figure 1), but when cooked they become a delicious preserve with a very sweet flavor that is very popular with people on the Ecuadorian coast. However, at present, studies related to their chemical composition and possible beneficial effect on health are scarce [13]. Recently, some reports have highlighted the composition and potential biological effects of different fruits consumed in Ecuador [4,5,6,8]. However, these studies are still limited given the country’s potential and traditional culture, which has used plants and fruits since ancient times for nutritional and medical purposes [14]. Therefore, the aim of this study was to determine the phytochemical profile of the nontraditional pechiche fruits as well as their potential role in preventing the oxidative damage in an in vitro model of human dermal fibroblast.

## 2. Materials and Methods 

### 2.1. Pechiche Fruits 

Pechiche fruits (*Vitex cymosa* Berteo ex Speng) were collected directly from the plant in the provinces of Guayas and El Oro, Ecuador, between December 2017 and February 2018, during their corresponding crop season. On two different occasions, two batches of 0.5 kg of fruits without damage and similar in terms of their degree of ripeness were randomly collected. The specimens were identified at the Jardín Botánico de Quito, Ecuador, using the reference vouchers available in the center’s herbarium. Fruits were freeze-dried, and then the exocarp and mesocarp were ground to a fine powder and stored at –20 °C until the analysis was performed. For phenolic acids analysis and mineral nutrient composition, the exocarp, the mesocarp and the whole fruit (without seeds) were analyzed separately.

### 2.2. Phenolic Acids Analysis

#### 2.2.1. Extraction of Phenolic Acids 

Phenolic acids were extracted according to previously reported methods [15] Sample powders (500 mg) were extracted with ethanol–water (80:20) and continuously shaken for 2 h in the dark at room temperature. Then samples were centrifuged twice (10 min, 5000 rpm) and filtered through a Whatman^®^ cellulose filters. The solid residue was re-extracted twice, and supernatants were combined. Finally, the extracts were dried under a vacuum and stored at −20 °C until further analysis.

#### 2.2.2. HPLC-DAD Analysis of Phenolic Acids Profile

Phenolic acids were identified and quantified using an Agilent HPLC system (Agilent Technologies Series 1260, Santa Clara, CA, USA) equipped with a quaternary pump (1260 Infinity G1312B) and a diode array detector (1260 Infinity G1315C DAD). The ZORBAX SB-C18 (3.5 µm, 4.6 × 150 mm) column was used as the stationary phase, and the mobile phase consisted of formic acid 0.1% (solvent A) and acetonitrile (solvent B) at a flow rate of 0.3 mL/min for 50 min. The elution was performed as follows: 0 min 80% A, 5 min 75% A, 15 min 60%, 20 min 45% A, 30 min 35% A, 35 min 35% A, 50 min 80% A. The identification of the phenolic acids was carried out by means of a comparison with the UV spectra, and the retention time of external standards at 280 and 330 nm. Phenolic acids were quantified using the corresponding calibration curve of the external standards and results were expressed as mg/100 g of fresh weight (FW).

### 2.3. Vitamin C and Carotenoid Content

Vitamin C was determined using an HPLC system (Agilent Technologies Series 1260, Santa Clara, CA, USA) equipped with a quaternary pump (1260 Infinity G1312B) and a diode array detector (1260 Infinity G1315C DAD). An Eclipse Plus C18 column (5 µm, 4.6 × 150 mm) was used as the stationary phase. The DAD was set at 245 nm, and elution was performed using KH_2_PO_4_ (50 mM, pH 2.5) in an isocratic gradient at a flow rate of 1 mL/min for 20 min. Ascorbic acid was used as a standard for the calibration curve (5–50 mg/L), and vitamin C content was expressed as mg of ascorbic acid per 100 g of FW of fruits (mg Vit C/100 g FW).

Carotenoids content was analyzed using the aforementioned HPLC system [6]. The DAD was set at 450 nm, methanol–isopropanol (35:65, v/v) was used for elution in an isocratic gradient at a flow rate of 1 mL/min for 15 min for elution using an Eclipse Plus C18 column (5 µm, 4.6 × 250 mm) as stationary phase. β-carotene (0.1–10 mg/mL) and lutein (0.1–50 mg/mL) were used for the calibration curve, and results were expressed as mg per 100 g of FW of fruits.

### 2.4. Inductively Coupled Plasma Optical Emission Spectrometry (ICP-OES) for Mineral Nutrient Composition

About 0.5 g of a fine powder of each sample was wet-oxidized with 10 mL of ultra-pure HNO_3_ (67% v/v) in a closed MARS 6 - Microwave Accelerated Reaction System (Smith Farm Road, Matthews, NC, USA). The analyses were carried out using an ICP-OES system (iCAP™ 7400 Duo ICP-OES Analyzer, Thermo Scientific™, Germany) equipped with Thermo Scientific™ Qtegra™ Intelligent Scientific Data Solution™ (ISDS) software (Thermo Scientific™, Waltham, MA, USA). Quality assurance and quality control were assessed using an ICP multi-element standard solution IV (23 elements, 1000 mg/L) (Merck KGaA, Germany). A Periodic table mix 1 for ICP TraceCERT^®^ (Merck KGaA, Germany) was employed, 33 elements were used as a standard and results were expressed as mg/Kg of FW.

### 2.5. Protective Effect of Crude Extract of Pechiche on Human Dermal Fibroblasts against Oxidative Damage

#### 2.5.1. Cell Line and Treatments

Primary human dermal fibroblasts isolated from adult skin (HDFa, ATCC^®^ PCS-201-012™, Manassas, VA, USA) were cultivated in a supplemented Eagle’s Minimum Essential Medium (EMEM) (10% fetal bovine serum, 2 mM of glutamine and 1% penicillin–streptomycin antibiotics (100 IU/mL penicillin and 100 µg/mL streptomycin)) at 37 °C in a humidified atmosphere with 5% CO_2_. The hydroalcoholic extract obtained previously (Section 2.2.1) was dried in a vacuum until all solvent was removed and the resulting crude extract (PCext) was resuspended in EMEM, to achieve the final concentration of 100 µg/mL. AAPH (2,2′-azobis(2-amidinopropane) dihydrochloride) (10 mM) diluted in EMEM was used to induce oxidative damage. A total of four experimental groups were formed as follows: (i) Ctrl—cells were incubated with the culture medium only; (ii) PCext—cells were incubated with pechiche crude extract for 24 h; (iii) AAPH—cells were incubated with AAPH (10 mM) for 24 h; and (iv) PCext + AAPH—cells were incubated with PCext for 24 h and then with AAPH (10 mM) for 24 h. The dose/time combination of the AAPH (10 mM) was determined through preliminary cytotoxicity assays and was selected according to its capacity to decrease cell vitality (~50%) compared to the control group (*p* ≤ 0.05) using the MTT [3-(4,5-diphenyl-tetrazolium bromide] colorimetric assay [16].

#### 2.5.2. Determination of Oxidative Damage Markers 

Intracellular ROS levels were determined using the 2′-7′-dichlorofluorescin diacetate (DCFH) kit (Merck, Germany), and results were expressed as arbitrary units of fluorescence intensity/µg cell protein. Cell adenosine triphosphate (ATP) levels were determined using the ATP Cell Titer Glo® assay (Promega, Madison, WI, USA), and results were expressed as fluorescence arbitrary units x 1000 cells.

For catalase, superoxide dismutase, glutathione activities, lipid peroxidation and protein oxidation, cells were lysed using the RIPA buffer and then stored at −80 °C until analyses. Catalase activity (CAT) [17] and superoxide dismutase (SOD) [18] were determined spectrophotometrically at 240 nm and 540 nm, respectively. For both assays, results were expressed as units per milligram of protein per minute (U/mg prot/min). Glutathione peroxidase (GPx), glutathione reductase (GR) and glutathione transferase (GST) [19] were also determined, and results were expressed as nmol of NADPH oxidized per milligram of protein per minute (nmol of NADPH oxidized/mg protein/min) for GPx and GR and nmol CDNB-GSH conjugate/mg protein/min for GST. Lipid oxidative damage was determined using the thiobarbituric acid-reactive substances (TBARS) [20] assay and hydroperoxides levels [21], and results were expressed as µM for both assays. For protein oxidative damage, protein carbonyl levels were determined through the 2,4-dinitrophenylhydrazine methods (DNPH method) [22], and results were expressed as nmol per milligram of protein (nmol /mg of protein). Protein content was determined by the Bradford protein assay [23].

### 2.6. Statistical Analyses

Statistical analyses were performed using IBM SPSS Statistic for Windows version 2.0 (Armonk, NY, USA). The samples were analyzed in triplicate, and results were reported as mean ± standard deviation (SD). Data between different groups were analyzed statistically using a one-way ANOVA and Tukey’s post hoc test. *p* ≤ 0.05 was considered as significant and *p* ≤ 0.01 highly significant.

## 3. Results and Discussion

### 3.1. Bioactive Compounds

Fruits are a rich dietary source of phenolic substances, of different origins and functions, which have been directly associated with health benefits such as anticancerous, antiviral and antibacterial properties [24]. Although studies on frequently consumed fruits are abundant, far fewer reports exist on the phenolic contents of nontraditional fruits and their biological properties. In this study, we evaluated the chemical composition and the protective effects against oxidative damage in vitro of the nontraditional pechiche fruit. Phenolic acids profile, carotenoids and vitamin C content of pechiche fruits are shown in Table 1. The compounds were identified on the basis of their UV obtained by HPLC-DAD, as well as their chromatographic behavior compared with external standards. Although a wide range of phenolic acid standards was used (15 in total), several peaks could not be identified, so additional studies are needed to further characterize the phenolic profile of this fruit. Up to five peaks could be assigned to phenolic acids, with homovanillic acid as the predominant one. Homovanillic acid and 4-hydroxyphenylacetic acid are a phenolic acid subclass of hydroxyphenylacetic acids that were previously reported in olive oil [25,26] and beer [27]. Homovanillic acid was also reported in pungent fruits [28] and tomatoes (*Solanum lycopersicum* L.) [29]. On the other hand, 3-(4-hydroxyphenyl) propanoic acid (phloretic acid) was also found in pechiche fruits. Phloretic acid is a hydroxy monocarboxylic acid consisting of propionic acid and has a 4-hydroxyphenyl group at the 3-position. It has a role to play as a plant metabolite and has also been previously reported in olive drupes [30]. The presence of homovanillic, phloretic acids and 4-Hydroxyphenylacetic acid in pechiche and olive products could be related to their phylogenetic closeness since both belong to the Lamiales order [31,32]. In fact, pechiche is also known as aceituno in Colombia and Venezuela, giving one an idea of the relationship between these species [18]. In relation to the other phenolic acids identified (*p*-coumaric and gallic acid), their presence and biological potential in fruits have been widely reported [24].

Total carotenoids and vitamin C content in pechiche fruits were analyzed and are shown in Table 1. The pechiche fruits showed low values of carotenoids compared with other Ecuadorian fruits [4,5,6] and tropical fruits from the region [33,34] and based on intake recommendations for carotenoids [35], whereas in the case of vitamin C, the values were within the previously reported range [6,33,34]. Pechiche fruits could be classified within the group of fruits with low vitamin C content (< 30 mg/100 g of FW) according to the classification previously proposed by Ramful et al. [36].

The values of vitamin C and carotenoids in pechiche and the values previously reported in Ecuadorian fruits could be different because of the fundamental altitude differences that exist between the ecosystems [37]. The reference values of Ecuadorian fruits previously reported correspond to those cultivated at more than 1000 m above sea level, whilst pechiche grows in ecosystems at sea level. Although these results provide an approximation to the phenolic content of pechiche fruits, additional analyses are necessary to deepen understanding of pechiche fruits’ chemical composition, especially their polyphenolic composition, such as flavonoids, which could more fully justify the biological results observed here. Our results represent, the first report on the chemical composition of pechiche fruits.

### 3.2. Nutrient Minerals Composition

The contents of micro- and macrominerals in pechiche fruits were also determined and are shown in Table 1. Contents of these groups of elements have been linked to the prevention of deficiency diseases that can put the development and life of human beings at risk [38], hence the importance of establishing the content of each of these in different foods consumed by different populations and ethnicities according to their customs. Phosphorus and potassium were the principal macrominerals found in pechiche fruits. Phosphorus is required for all known forms of life. It is fundamental to the structural framework of DNA and RNA, as well as for transporting cellular energy with adenosine triphosphate (ATP), necessary for every cellular process that uses energy [38]. Potassium was another important macromineral found in pechiche fruits, which has been related to beneficial effects on coronary heart diseases given that it decreases blood pressure and maintains an adequate Na^+^/K^+^ ratio [39]. Adequate dietary potassium is important for heart and bone health and reduces the risk of stroke and coronary heart disease [39]. Other important macrominerals identified in pechiche fruits were magnesium and calcium. Magnesium is fundamental for the retention of vitamin C and calcium in bones in order to maintain their structure and prevent osteoporosis and cardiovascular diseases [40]. Calcium is fundamental in human nutrition since it is involved in several vital processes such as muscle contraction, cell differentiation, neuronal activities and immune response [41].

Pechiche fruits also contain microminerals such as Fe^2+^, Mn^2+^ and Zn^2+^ that are involved in various metabolic processes vital to human health. Iron was a principal micromineral found in the fruits, followed by zinc (Table 1). Iron is fundamental in the respiratory process, as well as in DNA synthesis [42]. The trace element Zn is required for the function of over 300 enzymes and 1000 transcription factors. Zn is the second most abundant trace metal in human bodies after iron and is the only metal that appears in all enzyme classes [43]. In general, the pechiche fruits from the areas studied, which are the main areas where these fruits are harvested in Ecuador, proved to be a natural dietary source of important macro- and microelements that are indispensable for the human metabolism. Our results could be considered to encourage the consumption of this fruit in the region since there are still areas of these regions with high rates of malnutrition in different sectors of the population.

### 3.3. Protective Effect of Pechiche Extract against Oxidative Cytotoxic Damage in HDFa

Fruits have been proposed as an efficient dietary source of bioactive compounds with relevant protective effects against the damage caused by oxidative stress [24,44]. These effects are attributed mainly to their chemical composition and specifically to the presence of an important group of phyto-compounds such as phenolic acids, flavonoids, anthocyanins, carotenes and vitamin C [24]. In Ecuador, there are several nontraditional fruits consumed by a large sector of the population, such as pechiche, which have not been studied in order to clarify their potential health benefits. In this regard, in addition to studying its chemical composition, here we studied the ability of crude pechiche extract to protect HDFa against cytotoxic damage produced by oxidative stress. This experimental model is widely used to evaluate several biological aspects of food extracts, drugs and natural compounds, allowing results to be obtained with high reproducibility, reliability and sensitivity, especially when published data on a particular topic are scarce. Moreover, the use of HDF as an in vitro model could also justify the use of pechiche in the formulation of cosmetic products with proven antioxidant properties.

**Figure 2:**
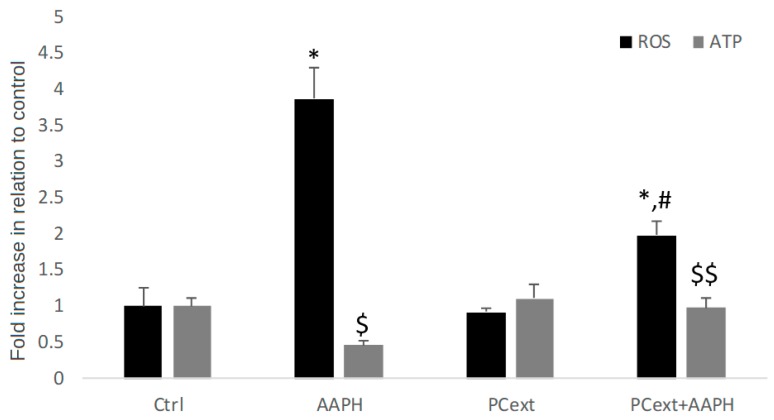
Intracellular reactive oxygen species (ROS) and ATP levels in human dermal fibroblasts (HDFa), showing Crtl (Control cells), treatment with crude pechiche extract (PCext), treatment with the stressor AAPH (AAPH) and pretreatment with crude pechiche extract and then stressed with AAPH (PCext-AAPH). Results are reported as mean ± SD of three experiments. * *p* ≤ 0.05, significant differences compared to control and ^#^ *p* ≤ 0.05, significant differences between AAPH and PCext-AAPH groups for intracellular ROS levels; ^$^ *p* ≤ 0.05, significant differences compared to control and ^$$^ *p* ≤ 0.05, significant differences between AAPH and PCext-AAPH groups for ATP levels.

AAPH is a free-radical-generating azo compound used as an oxidant in in vitro models because of its capacity to oxidize in small molecules by using its ability to initiate oxidation reactions via both nucleophilic and free radical mechanisms that are able to induce cell apoptosis and therefore death [45]. When cells were incubated with AAPH, a significant increase (*p* ≤ 0.01) in intracellular ROS levels was observed as compared to the control. The positive effect of PCext on cell viability may be due, in part, to the improvement in intracellular ROS levels in preincubated cells with PCext (PCext + AAPH). In fact, when cells were preincubated with PCext, the intracellular ROS values significantly decreased (*p* ≤ 0.05) in comparison to the AAPH-stressed group (AAPH group) (Figure 2). A similar behavior was observed when the intracellular content of ATP was determined as a marker of mitochondrial damage in cells. The AAPH treatment significantly decreased (*p* ≤ 0.05) the intracellular content of ATP in comparison to the control group, showing significant damage to the integrity and mitochondrial functioning of the cells (Figure 2). However, when HDFa were preincubated with PCext and then stressed, ATP levels significantly improved (*p* ≤ 0.05) in comparison to the AAPH group. The results shown here agree with a large number of reports that have shown the protective effect of the polyphenols present in fruits against free-radical-mediated cell damage [24,44,46]. In particular, the results presented here add to the previous reports that show similar properties in other fruits consumed in Ecuador, such as capulí [5], and wild Andean blackberry and blueberry [4].

Several studies have demonstrated the positive effects of plant bioactive compounds, such as polyphenols, in modulating the activity of diverse antioxidant enzymes (GPx GR, GST, SOD and catalase) by reducing the damage induced by AAPH and other different stressors and restoring conditions similar to control levels in HDFa [47,48,49]. In our study, the capacity of PCext to protect the antioxidant enzymes and lipids and proteins against oxidative damage was also demonstrated and the results are shown in Table 2. When HDFa were incubated with AAPH, the activity of the antioxidant enzymes CAT, SOD, GPx, GR and GST was significantly affected (*p* ≤ 0.01) as compared to the control. Similarly, the values of TBARS and lipid hydroperoxides, as well as protein carbonyl levels, as markers of lipids and proteins damage, respectively, increased dramatically (*p* ≤ 0.01) compared with the control group. However, when cells were preincubated with PCext, the activity of the antioxidant enzyme CAT significantly improved (*p* ≤ 0.05) in comparison to the AAPH-treated group, while regarding the activity of the enzymes SOD, GPx, GR and CST, it improved significantly (*p* ≤ 0.01) compared to the AAPH-treated group. Similarly, the oxidative damage markers to lipids (*p* ≤ 0.05) and proteins (*p* ≤ 0.01) were significantly lower compared to the AAPH-treated group. Lipid peroxidation is a free-radical-mediated chain reaction involving several types of free radicals, which could be counteracted through enzymatic means or by the free-radical-blocking activity exerted by the antioxidant compounds [49]. Protein oxidation, measured as an increase in carbonyl groups, could be used to determine accumulation of oxidative damage to proteins in the long term, as in aging studies [21]. Therefore, according to the results presented here, one could hypothesize that the capacity of pechiche extract to protect lipids and proteins against oxidative damage could be related to the capacity of pechiche antioxidant compounds to block free radicals and activate antioxidant enzymes.

## 4. Conclusions

Through scientific evidence, the results presented here reinforce the beneficial health aspects of nontraditional fruits that have been poorly studied. Our findings constitute the first report about Ecuadorian pechiche fruits’ antioxidant properties and possible mechanisms. This study’s new evidence could serve as a scientific basis to justify pechiche fruits’ health potential as a source of bioactive compounds and nutrient minerals. The proposed biological effects include the ability to activate the antioxidant enzymes response, such as SOD and CAT, and the protection of lipids and proteins against oxidative damage; therefore, our results could represent the basis to perform new research to develop food supplements or cosmetic products with proven antioxidant properties.

## Figures and Tables

**Table 1:** Phenolic acids profile, carotenoids, vitamin C and mineral nutrients content of pechiche fruits.

Analysis	Values
Phenolic acids (mg/100 g of FW)	
Gallic acid	22.60 ± 12.74
3-(4-Hydroxyphenyl)propanoic acid	24.11 ± 2.62
*p*-Coumaric acid	4.87 ± 0.01
Homovanillic acid	42.92 ± 1.34
4-Hydroxyphenylacetic acid	12.52 ± 1.55
Carotenoids (µg/g of FW)	
β-carotene	0.78 ± 0.02
Lutein	22.7 ± 3.84
Vitamin C (mg/100 g of FW)	25.56 ± 1.14
Macrominerals (mg/Kg of FW)	
Na	197.13 ± 14.15
K	852.28 ± 21.05
Ca	546.56 ± 26.18
Mg	653.65 ± 32.25
P	1550.05 ± 102.36
Microminerals (mg/Kg of FW)	
Fe	28.87 ± 3.16
Mn	6.25 ± 1.16
Zn	9.94 ± 1.28

Results are reported as mean ± SD of three determinations.

**Table 2:** Biomarkers of oxidative damage in HDFa.

Markers	Ctrl	AAPH	PCext	PCext + AAPH
CAT (U/mg prot/min)	46.85 ± 1.64	18.74 ± 2.41 **	42.06 ± 3.11	32.11 ± 2.66 ^#^
SOD (U/mg prot/min)	78.22 ± 3.44	32.98 ± 2.87 **	81.74 ± 5.84	58.51 ± 3.21 ^##^
GPx (nmol mg prot/min)	271.82 ± 22.47	83.41 ± 4.20 **	282.44 ± 36.21	176.52 ± 10.84 ^##^
GR (nmol mg prot/min)	282.65 ± 18.41	72.68 ± 9.41 **	286.84 ± 21.46	188.32 ± 9.23 ^##^
GST (nmol /mg protein/min)	564.51 ± 36.87	226.12 ± 14.94 **	571.68 ± 26.13	396.44 ± 21.03 ^##^
TBARS (µM)	5.22 ± 0.84	9.69 ± 0.54 **	5.86 ± 0.64	6.44 ± 0.31 ^#^
Lipid hydroperoxides (µM)	66.54 ± 7.12	167.51 ± 10.41 **	68.71 ± 6.91	71.84 ± 4.52 ^#^
Protein carbonyl (nmol/mg prot)	0.82 ± 0.08	2.01 ± 0.21 **	0.76 ± 0.04	1.12 ± 0.04 ^##^

** *p* ≤ 0.01, significant differences compared to control and ^#^ *p* ≤ 0.05, ^##^ *p* ≤ 0.01 significant differences between AAPH and PCext-AAPH group. CAT, Catalase; SOD, Superoxide dismutase; GPx, Glutathione peroxidase; GR, Glutathione reductase; GST, Glutathione transferase; TBARS, Thiobarbituric acid reactive substances.
